# Influence of Pterostilbene on Gene Expression in Liver Cancer: An In Silico Analysis

**DOI:** 10.7759/cureus.53098

**Published:** 2024-01-28

**Authors:** Monisha Prasad, Silambarasan Tamil Selvan, Rajeshkumar Shanmugam

**Affiliations:** 1 Centre for Global Health Research, Saveetha Medical College and Hospital, Saveetha Institute of Medical and Technical Sciences, Chennai, IND; 2 Nanobiomedicine Lab, Centre for Global Health Research, Saveetha Medical College and Hospital, Saveetha Institute of Medical and Technical Sciences, Chennai, IND

**Keywords:** therapeutic targets, network pharmacology, differentially expressed genes (degs), gene expression omnibus (geo) datasets, pterostilbene, liver cancer

## Abstract

Background

Liver cancer, in particular, is a serious threat to global health and has few viable treatments. One natural molecule that shows potential in cancer therapy is pterostilbene, especially for hepatocellular carcinoma (HCC). The molecular details of pterostilbene’s interactions with liver cancer are uncovered in this study using an in silico method.

Methodology

This study determines the differentially expressed genes (DEGs) in HCC and the way pterostilbene affects them using data from Gene Expression Omnibus (GEO) datasets. To identify the intricate linkages and possible treatment targets, network pharmacology, protein-protein interaction (PPI) analysis, and pathway enrichment investigations were performed.

Results

The study revealed complex relationships between pterostilbene and liver cancer, identified important DEGs in HCC, and showed enriched pathways. Pterostilbene shows promise as a target for therapeutic approaches in HCC due to its modulation of important signaling pathways.

Conclusions

This work offers an extensive knowledge of pterostilbene’s potential in liver cancer, despite intrinsic computational limitations. In addition to the importance of experimental validation, the pathways and DEGs that have been found provide insightful information for future investigation, highlighting the ongoing research that is necessary to create targeted therapeutics for HCC.

## Introduction

Liver cancer is the primary cause of cancer-related mortality and the fifth most frequent malignancy globally. Among the spectrum of liver malignancies that includes cholangiocarcinoma (bile duct cancer), angiosarcoma, hemangiosarcoma, and hepatoblastoma, hepatocellular carcinoma (HCC) accounts for 75-85% of cases. This is a significant global health concern. In 2020, liver cancer was diagnosed in 905,700 cases worldwide and cost the lives of 830,200 people. It was among the top three causes of cancer-related mortality in 46 countries. It is anticipated that there will be a more than 50% increase in liver cancer-related deaths and new cases by 2040 [1]. Persistent infection with the hepatitis B and C viruses is one of the risk factors linked to the development of HCC. In addition to standard chemotherapy, HCC is treated with a range of curative methods such as liver transplantation and surgical excision. Up to 80% of people have been observed to experience an HCC recurrence within five years. Therefore, it is essential to design medications that guarantee patient safety while also enhancing the efficacy of HCC therapy. Regretfully, adverse effects from current treatments, particularly those that occur with continuous usage, have been shown to include gastrointestinal toxicity, hepatotoxicity, cardiotoxicity, and neurotoxicity. Patients receiving chemotherapy and radiation therapy for a variety of malignancies, including HCC, frequently have these side effects. Therefore, it is essential to keep researching anticancer drugs derived from plants to reduce the side effects of chemotherapy and make it safer [2].

Combinations of genetic and epigenetic modifications define HCC, a complicated and multifaceted biological process. Major advancements in our understanding of the critical tumor suppressor and oncogenic pathways linked to HCC are essential [3]. To tackle present remedy issues, it is critical to identify therapeutic solutions, as previously mentioned. Determining novel targets for treatment development can be facilitated by exploring the diverse cell signaling pathways associated with tumor etiology. In contrast to traditional cytotoxic drugs that have a wide range of effects, targeted therapies precisely alter elements that contribute to the development of tumors [4].

Innovative treatments for HCC have been found as a result of the precise targeting of vital pathways, which include the receptor tyrosine kinase pathways, the Ras mitogen-activated protein kinase (Ras/Raf/MAPK), the phosphatidylinositol 3-kinase (PI3K)/ protein kinase B Akt/mammalian target of rapamycin (mTOR), the Wingless/Integrated (Wnt)/β-catenin signaling pathway, the ubiquitin/proteasome degradation pathway, and the hedgehog signaling pathway [5,6].

Pterostilbene, a natural stilbene compound closely related to resveratrol, has demonstrated potent anticancer properties, exhibiting effectiveness in various cancer types, including bladder, breast, colon, liver, and prostate cancer [7-11]. Derived from sources such as blueberries, pterostilbene is well-known for its antioxidant, anti-inflammatory, and anticancer activities. In the context of HCC, pterostilbene has proven its significance by targeting key signaling pathways involved in tumorigenesis, such as nuclear factor kappa-light-chain-enhancer of activated B cells (NF-κB), tissue-type plasminogen activator (TPA)-induced matrix metalloproteinases (MMP-9), vascular endothelial growth factor (VEGF), TPA-induced protein kinase C (PKC), mitogen-activated protein kinase (MAPK), p38, PI3K, and Akt [10]. However, there is a current gap in data regarding pterostilbene’s properties concerning inflammatory signaling that regulates liver cancer. Notably, inflammation plays a pivotal role in HCC progression, with the Wnt/β-catenin signaling pathway being a key player in its development. Aberrant activation of the Wnt signaling pathway is a frequent observation in HCC, contributing to increased cell proliferation and survival. In this study, pterostilbene has been identified as an inhibitor of the Wnt/β-catenin pathway, effectively suppressing the growth of HCC cells [10,12]. This finding highlights pterostilbene’s potential as a valuable therapeutic agent in HCC treatment. The present investigation employed a network pharmacology approach to construct an extensive *component-targets-pathway* network, Kyoto Encyclopaedia of Genes and Genomes (KEGG) pathway analysis, and gene ontology (GO) enrichment analysis. This comprehensive strategy aims to unravel the complex mechanisms underlying pterostilbene’s potential as a therapeutic option for liver cancer.

## Materials and methods

Data collection

Data from the Gene Expression Omnibus (GEO), managed by the National Center for Biotechnology Information, is a freely accessible collection housing varied and multidimensional information sourced from microarray experiments and next-generation sequencing technologies. Two sets of gene expression data (GSE84402, GSE46408) pertaining to liver cancer were accessed and retrieved from the GEO repository. GSE84402 contained six sets of matched normal and HCC samples, while GSE46408 encompassed 12 pairs of normal tissue samples contrasted with HCC tissues.

Microarray data pre-processing

The Series Matrix Files for GSE84402 and GSE46408 were acquired from the GEO database to facilitate further analysis. Before analysis, probe data within each dataset were converted into standard gene symbols. This step streamlines the interpretation of microarray results by aligning gene identifiers to a common nomenclature. To ensure consistency and eliminate potential technical biases, all four datasets underwent normalization using the robust multi-array average method in R software (version 2.6.0). This normalization process standardizes gene expression data across datasets, placing them on a uniform scale and distribution. By equalizing the data in this manner, any systematic variations between samples or experimental conditions are minimized, enabling more dependable comparisons and analysis across the datasets.

Identification of differentially expressed genes (DEGs) in HCC/pterostilbene datasets

In this study, the colorectal cancer datasets were analyzed using GEO2R to pinpoint DEGs. The volcano plot generated through GEO2R, available at the provided link, illustrates statistical significance (p-value) on the y-axis and fold change in gene expression on the x-axis. This visualization assists in identifying genes exhibiting significant expression alterations. The criteria for defining DEGs involved a p-value cutoff of <0.01 and an absolute log fold change >1. To supplement this analysis, gene cards specific to liver cancer targets and the Comparative Toxicogenomics Database (CTD) for the compound pterostilbene were utilized. Additionally, FunRich V3.1.3 software was employed to visualize the overlap and disparities in DEGs across these datasets. The Venn diagram generated by FunRich offers a clear representation of common molecular targets or pathways shared among the datasets. This aids in understanding how key genes or pathways connect with HCC, facilitating insights into their interplay.

Protein-protein interaction (PPI) network interaction and module analysis of DEGs in HCC

This study explored the PPI network involving efficient compound interactions using DEGs sourced from HCC datasets and the Search Tool for Interactions of Chemicals (STITCH) database. A combined score >0.08 was deemed significant for the interactions, denoting the reliability of the protein associations identified. The resulting DEGs were employed to construct and visualize the PPI network using Cytoscape software (version 3.5.1; http://www.cytoscape.org). In this constructed PPI network, the connections between proteins were represented by edges, with widths reflecting the strength of these protein interactions based on the combined score. The study identified Hub genes, characterized as nodes with a degree >10, indicating their significance within the network. To uncover highly interconnected gene clusters within the PPI network, the study utilized the Molecular Complex Detection (MCODE) and CytoHubba plugins of Cytoscape. By configuring parameters such as a node score cut-off of 0.2, a k-core value of 2, and a maximum depth of 100, these plugins detected and isolated core modules or clusters of closely linked genes. These gene clusters are likely representative of functional units or pathways within the network.

GO and pathway enrichment analysis

In this study, the investigation extended to functional and pathway enrichment analysis, a critical step in deciphering the biological relevance of identified DEGs and gene clusters. To gain deeper insights into the biological functions and enriched pathways associated with upregulated and downregulated hub genes, the g:Profiler database (https://biit.cs.ut.ee/gprofiler/) was utilized. This tool offers systematic and standardized annotations, categorizing genes into molecular functions (MFs), cellular components (CCs), and biological processes (BPs). Moreover, KEGG pathway analysis was conducted to pinpoint pathways exhibiting notably enriched associations with the identified DEGs. For pathway crosstalk analysis, the study applied specific thresholds: a Benjamini-Hochberg adjusted p-value <0.05, and a combination of the Jaccard coefficient (50%) and overlap coefficient (50%) >0.5, considered statistically significant. By scrutinizing the DEGs within particular pathways, their potential roles in pivotal biological processes and regulatory pathways were elucidated.

## Results

Identification of DEGs in HCC

**Figure 1:**
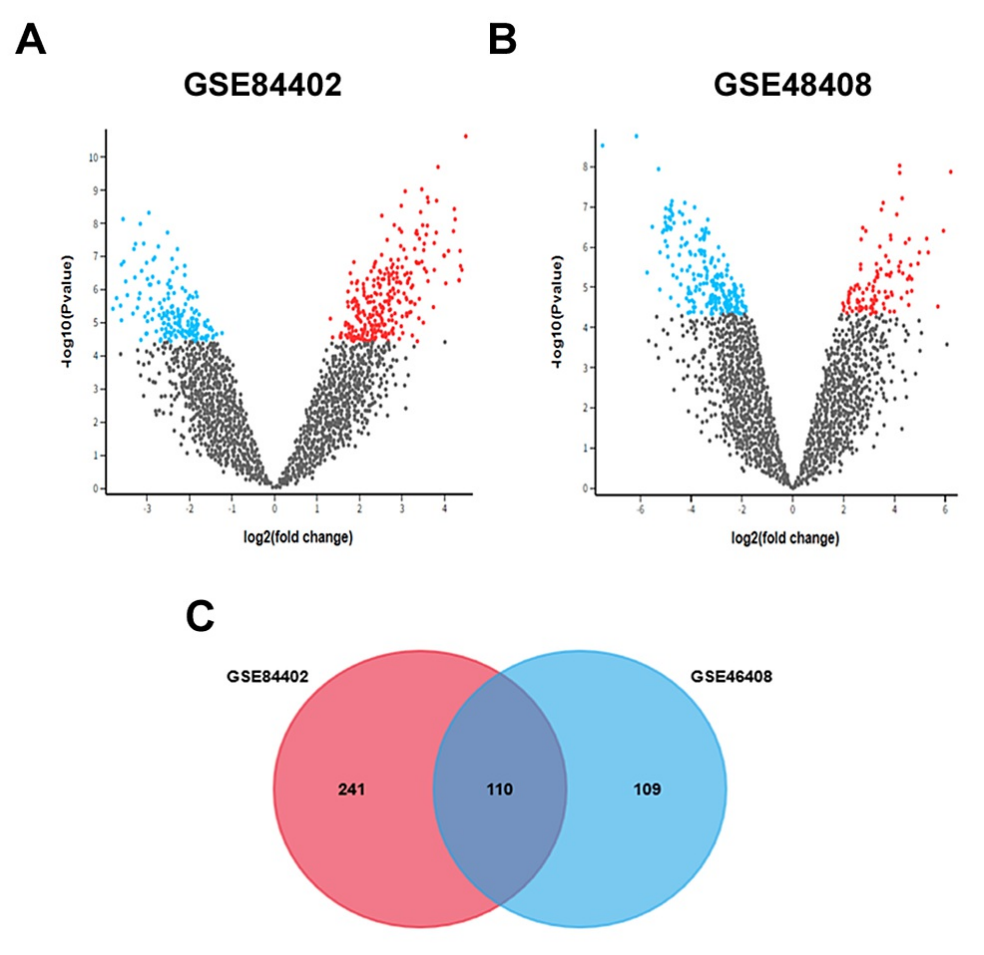
A total of 351 and 219 differentially expressed genes (DEGs) (adjusted p-value <0.01, log fold change >1) were found in the GSE84402 (A) and GSE46408 (B) datasets, respectively. A total of 110 consistently upregulated DEGs are shown in Venn diagram (C), which was created using FunRich_V3.

A total of 351 and 219 DEGs were identified from the HCC cancer datasets GSE84402 and GSE46408, respectively. Using GEO2R analysis with the Limma package and applying a threshold of adjusted p-value <0.01 and log fold change >1, volcano plots (Figures 1A, 1B) were generated for each dataset. The results prominently indicated an upregulation of the majority of DEGs in liver cancer samples. To pinpoint common DEGs across the two HCC datasets, an intersection analysis was performed utilizing FunRich_V3 software. Figure 1C illustrates the expression patterns of the shared upregulated DEGs identified from GSE84402 and GSE46408 datasets. The Venn diagram visually depicts the overlapping DEGs among the COAD datasets, revealing that 110 genes are significantly upregulated in liver cancer datasets.

Identification of DEGs involved in pterostilbene in liver cancer

**Figure 2:**
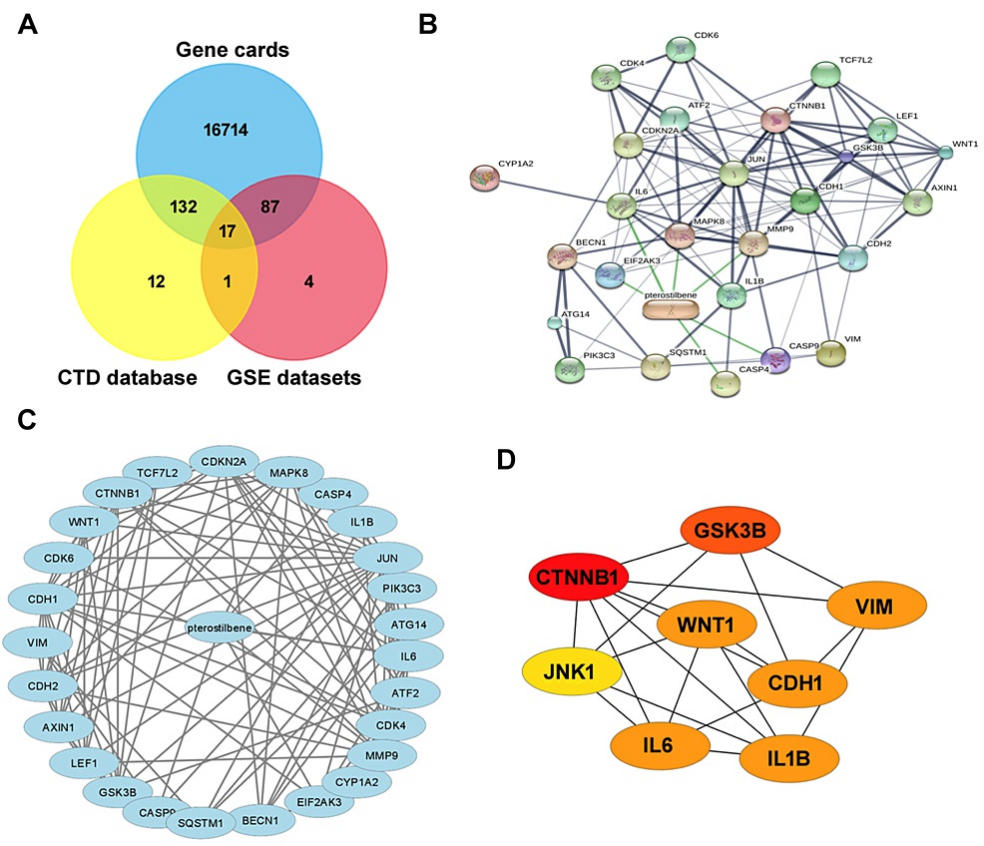
Interactions between pterostilbene and liver cancer Venn diagram (A) shows 17 common targets; network (B) for 114 upregulated differentially expressed genes (DEGs) and 66 downregulated DEGs is produced by STITCH analysis (threshold >0.4). For upregulated DEGs, protein-protein interaction network (C) shows 22 nodes and 114 edges. CytoHubba provides insights into hepatocellular carcinoma carcinogenesis by identifying important genes (D) via GO and pathway analysis.

In Figure 2A, the Venn diagram demonstrates the overlapping targets involved in interactions between pterostilbene and liver cancer. A total of 17 targets were common among the analyses conducted using Genecards, the CTD database, and GSE datasets. To explore the pathways and biological functions of these targets in HCC concerning pterostilbene, the STITCH database was employed with a confidence score threshold of >0.4. The resulting network was visualized using Cytoscape software (Figure 2B). Separating 114 upregulated DEGs and 66 downregulated DEGs, distinct PPI networks were mapped. The upregulated DEG network depicted 22 nodes and 114 edges, as presented in Figure 2C. Moreover, CytoHubba identified top-ranking genes based on high degree, radiality, and betweenness (Figure 2D). These core modules and genes were selected for further analysis using GO and pathway analysis, aiming to gain deeper insights into their functional roles in HCC tumorigenesis.

Identification of GO and KEGG pathway enrichment analysis of upregulated genes

**Figure 3:**
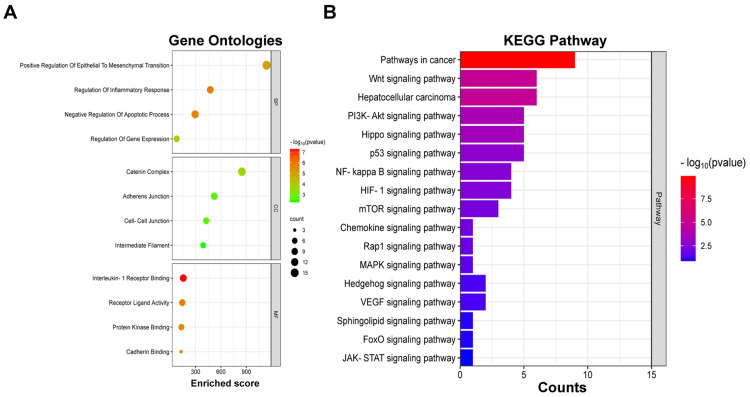
g:Profiler analysis of elevated differentially expressed genes in hepatocellular carcinoma indicates participation in important molecular and biological processes (A). The identification of probable processes in liver tumorigenesis is suggested by KEGG pathway enrichment, which shows relationships with Wnt signaling, pathways in cancer, and hepatocellular carcinoma (B).

The GO analysis conducted through the g:Profiler web server provided crucial insights into the route enrichment and functional annotation of upregulated DEGs in HCC. The study revealed significant enrichment of upregulated DEGs in various biological processes, such as positive regulation of epithelial to mesenchymal transition, regulation of the inflammatory response, and negative regulation of the apoptotic process. Notably, molecular functions such as IL-1 receptor binding, cadherin binding, and protein kinase binding showed high enrichment. Changes in cellular components were observed in structures such as the catenin complex, adherens junction, and cell-cell junction (Figure 3A). Furthermore, the KEGG pathway enrichment test unveiled significant associations between the upregulated DEGs affected by pterostilbene in HCC and multiple pathways. There was a pronounced enrichment in pathways such as the Wnt signaling pathway, pathways in cancer, and particularly those regulated in HCC (Figure 3B). These findings shed light on potential molecular mechanisms involved in liver tumorigenesis, suggesting that targeting these potent DEGs affected by pterostilbene could offer deeper insights into combating the illness.

## Discussion

The pharmacological networks linked to pterostilbene in liver cancer were thoroughly investigated in this study. To acquire a basic grasp of the compound’s effects and methods of action, we started by gathering relevant data from publications and molecular databases. Then, using bioinformatics techniques, we built and examined extensive pharmacological networks, deciphering complex signaling pathways, gene expression profiles, and PPIs that are impacted by pterostilbene.

We found particular therapeutic targets within these networks, including important molecular players such as hub genes, enzymes, and receptors that are affected by pterostilbene. Concurrently, we investigated the pathways that the chemical impacts, providing insight into its wider effects on intracellular signaling cascades and metabolic activities [13]. Our study’s robustness is strengthened by the interconnectedness of our findings, which provide a comprehensive understanding of the potential therapeutic benefits of pterostilbene in liver cancer. These findings not only have the potential to be clinically significant for targeted therapeutics and biomarker identification but they also open the door for additional studies to confirm the applicability of these pharmacological networks in practical applications.

The finding of 219 and 351 DEGs from the GSE46408 and GSE84402 datasets, respectively, not only makes a substantial contribution to the understanding of HCC but also carefully connects genetic discoveries with the state of clinical practice. This work highlights conserved molecular aberrations in liver cancer by matching known HCC gene expression patterns. A distinct molecular signature consisting of 110 common increased DEGs is being investigated for possible involvement in important signaling pathways such as Wnt, MAPK, and PI3K-Akt [14]. The study emphasizes techniques including surgical resection, biannual ultrasonography surveillance, and liver transplantation for cirrhotic patients, acknowledging the therapeutic importance of these findings within the larger framework of liver cancer research. The necessity for continuous research to improve clinical procedures and the management of HCC is highlighted by common methods such as image-guided ablation, chemoembolization, and systemic therapy [15,16].

The detailed examination presented in Figure 2 sheds light on the complex relationships between pterostilbene and liver cancer, especially HCC. The study establishes a strong basis for investigating the complex effects of pterostilbene by identifying 17 common targets among Genecards, the Comparative Toxicogenomics Database, and GSE datasets. Using the STITCH database revealed the routes and functional roles in HCC, which are shown graphically as a complex network (Figure 2B). Subsequent exploration of 114 upregulated and 66 downregulated DEGs culminated in distinct PPI networks (Figure 2C). The upregulated DEG network, with 22 nodes and 114 edges, was further scrutinized using CytoHubba to identify critical nodes in Figure 2D. This intricate molecular interplay, as suggested by McCormack and McFadden [17], aligns with pterostilbene’s antioxidant potential, underscoring its promising role in modulating key molecular interactions in HCC.

These core modules and top-ranking genes underwent GO and pathway analysis to gain insights into their roles in HCC tumorigenesis, unraveling the biological processes and pathways influenced by pterostilbene in liver cancer. In correlation with existing research, pterostilbene demonstrates its ability to alter gene expression, inhibit phosphorylated signal transducer and activator of transcription 3, and reduce tumor growth in pancreatic cancer [18]. Moreover, its superior bioavailability compared to resveratrol after oral administration has been highlighted [19,20]. Together, these findings, coupled with our results, emphasize the diverse potential of pterostilbene in shaping cancer dynamics, providing valuable insights for future therapeutic investigations.

This research delves deeper into the pharmacological networks that pterostilbene influences in liver cancer, with a particular emphasis on HCC. The study uses bioinformatics techniques to uncover how pterostilbene affects intricate signaling cascades, gene expression levels, and PPIs. The robustness of the investigation is strengthened by the identification of therapeutic targets, such as hub genes, enzymes, and receptors. The examination of genes with differential expression from the GSE46408 and GSE84402 datasets provides important new understandings of HCC and its therapeutic consequences. The research highlights unique molecular signatures and conserved molecular aberrations, connecting them to important signaling networks. The study emphasizes the necessity of continuing research to improve clinical management by acknowledging established clinical methods for HCC. The present study’s comprehensive analysis highlights the complex connections between pterostilbene and liver cancer and lays a strong foundation for additional research. In summary, the results shed light on the various and auspicious functions of pterostilbene in influencing the dynamics of liver cancer and provide a significant understanding of prospective treatment strategies.

Limitations

Recognizing the inherent limitations is crucial, even though our study uses a strong in silico technique to clarify the possible therapeutic influence of pterostilbene in liver cancer. Experimental validation is necessary to confirm the anticipated interactions, as the findings rely on bioinformatics analyses. A thorough understanding of pterostilbene’s therapeutic success in HCC would require additional in vitro and in vivo investigations, as the study mostly focuses on computational predictions and the biological effects of the drug may differ.

## Conclusions

This study investigated the pharmacological networks in liver cancer, specifically HCC, that are impacted by pterostilbene. The study identified possible therapeutic targets by using bioinformatics to uncover complex signaling networks, gene expression profiles, and PPIs impacted by pterostilbene. Understanding the molecular landscape of HCC was facilitated by analyzing DEGs from the GSE46408 and GSE84402 datasets. Consistent molecular aberrations and a unique molecular signature associated with important pathways are highlighted by the results, underscoring the necessity of continued research to improve therapeutic approaches. Pterostilbene’s potential in liver cancer is better understood overall owing to this research, which paves the way for future studies on targeted treatments and clinical applications.
